# Lower Body Symmetry and Running Performance in Elite Jamaican Track and Field Athletes

**DOI:** 10.1371/journal.pone.0113106

**Published:** 2014-11-17

**Authors:** Robert Trivers, Bernhard Fink, Mark Russell, Kristofor McCarty, Bruce James, Brian G. Palestis

**Affiliations:** 1 Graduate Program in Ecology and Evolution, Rutgers University, New Brunswick, New Jersey, United States of America; 2 Courant Research Centre, Evolution of Social Behavior & Institute of Psychology, University of Göttingen, Göttingen, Germany; 3 Faculty of Health and Life Sciences, Northumbria University, Newcastle-upon-Tyne, United Kingdom; 4 Department of Psychology, Faculty of Health and Life Sciences, Northumbria University, Newcastle-upon-Tyne, United Kingdom; 5 MVP Track and Field Club, University of Technology, Kingston, Jamaica; 6 Department of Biological Sciences, Wagner College, Staten Island, New York, United States of America; University of Paris 13, France

## Abstract

In a study of degree of lower body symmetry in 73 elite Jamaican track and field athletes we show that both their knees and ankles (but not their feet) are–on average–significantly more symmetrical than those of 116 similarly aged controls from the rural Jamaican countryside. Within the elite athletes, events ranged from the 100 to the 800 m, and knee and ankle asymmetry was lower for those running the 100 m dashes than those running the longer events with turns. Nevertheless, across all events those with more symmetrical knees and ankles (but not feet) had better results compared to international standards. Regression models considering lower body symmetry combined with gender, age and weight explain 27 to 28% of the variation in performance among athletes, with symmetry related to about 5% of this variation. Within 100 m sprinters, the results suggest that those with more symmetrical knees and ankles ran faster. Altogether, our work confirms earlier findings that knee and probably ankle symmetry are positively associated with sprinting performance, while extending these findings to elite athletes.

## Introduction

Trivers et al. [1] showed that Jamaican eight year olds of both sexes with more symmetrical knees were better sprinters 14 years later in both the 100 and the 200 m dashes, while symmetry of the upper body and feet did not predict sprinting speed, and ankle symmetry sometimes seemed to have a minor positive effect on sprinting speed. There are two separate reasons why we might expect symmetry of lower body traits to be positively associated with sprinting speed. Symmetry is inherently more efficient in races–it is less physically demanding and thus saves energy. This is presumably why the lower body asymmetry of Jamaican children 8.2 years of age is 1/3^rd^ that of upper body asymmetry [2]. Walking and running are inherently symmetrical and should favor symmetrical traits associated with them while upper body movements may or may not be symmetrical (vide, laterality) [3], [4].

On the other hand, it has been known since the 1950’s from experimental work on *Drosophila* that stress during early development is associated with greater adult bodily asymmetry, as is genetic inability to deal with the stress (e.g., inbred vs. outbred). This led to the notion that fluctuating asymmetry (FA) – deviations from bilateral symmetry in paired traits, randomly distributed to the left and right side – is a measure of developmental instability, i.e., the inability of an organism genetically to buffer the system against stressors to achieve the optimal state, namely symmetry itself [5]. The key is that if a population shows true FA then it can be presumed to be attempting to be symmetrical, so that failure to do so is a measure of failure to reach–in the face of developmental perturbations–the phenotype that the genotype is aiming for. Once it was discovered that females in a variety of birds, insects and mammals preferred symmetrical males as mating partners, even in species lacking male parental investment, it became obvious that body symmetry must be positively associated with genetic quality–a resistance from disease, for example [6], and many other advantageous traits [7].

To test for associations between symmetry and athletic performance, we compared lower body symmetry (knees, ankles and feet) in a control sample of Jamaicans measured in the countryside, carefully matched to a sample of elite Jamaican athletes measured in Kingston (all members of the MVP Track and Field Association). Among the elite athletes we also obtained performance–best times in preferred events against world records–to see if variation in peak sprinting times was in part predicted by variation in lower body symmetry.

In this paper we show that elite athletes have markedly more symmetrical knees than countryside controls and also more symmetrical ankles, while symmetry of feet does not differ between the two. We further demonstrate that within elite sprinters, performance shows striking and consistent positive associations with both knee and ankle symmetry. To wit, taking all athletes together, knee symmetry predicts sprinting ability while ankle symmetry almost does so. By adding covariates, such as weight and age, knee and ankle symmetry remain as marginally significant predictors of athletic success at the highest level of elite performance. Considering only the 100 m sprinters (n = 32) we find positive correlations between degree of knee and ankle symmetry and world performance. It also seems noteworthy that overall symmetry decreases among those who run 200 m, 400 m or 800 m races, as if adapted to or caused by frequent left-hand turns.

## Methods

### Subjects

We recruited two groups of subjects: elite Jamaican track and field athletes and controls from the Jamaican countryside. The elite athletes were all members of the MVP Track and Field Club working out of University of Technology in Kingston. We were able to get a nearly complete sample of club membership (73 of 77). There are exclusive criteria for being members of MVP and it is highly sought after by up and coming Jamaican athletes. International Association of Athletics Federations (IAAF) scores allow comparisons across disciplines and genders [8] (www.iaaf.org) and we used these scores, which we calculated based on personal bests, as our measure of performance. Approximately 90% of MVP club members have IAAF scores above 1000, and 6 of our subjects have remarkably high scores (≥1200). Although their training all included sprinting, the athletes’ best events varied, and in addition to comparing athletes and controls, we also compared symmetry of athletes whose specialties differed.

Control subjects consisted of individuals recruited in the countryside through word of mouth by someone who knew the community very well and had worked with the Jamaican Symmetry Project [2] since its inception in 1996. This person was given an exact list of the ages and sexes of the elite athletes we would be measuring in Kingston, so she could recruit a matching sample, which she did exactly. We then invited additional subjects who were within the age range of the elite athletes to appear and be measured, except that we discouraged all overweight individuals (which were overwhelmingly women) since we doubted there were many overweight elite athletes. The final number of control subjects was 116.

Elite athletes and controls were of very similar age (athletes: mean+/−SD = 23.0+/−3.2; controls 23.0+/−3.6; t_187_ = 0.14, p = 0.89). There is also no difference in variance in age between groups (Levene’s test, F = 1.68, p = 0.20) and the ranges are as follows: elite 17.13–31.95; control 17.21–32.46.

### Measurement of traits

Before traveling to Jamaica the three measurers met, and each measurer was exclusively allocated one trait to eliminate between-measurer effects. There were two sessions, each of approximately 6 hours, in which the measurement points for knees, ankles and feet were agreed upon and preliminary repeatability for each trait was established. Measurement points were the same as adopted in the Jamaican Symmetry Project in 1996 [2] and thus match the landmarks used in Trivers et al. [1].

When conducting the study in Jamaica, we measured the traits twice per side for each individual (second foot measurements missing for one subject). Repeated measurement is standard in FA studies, because the differences between sides are often so small that they can be similar in magnitude to measurement error [9]–[11]. Only by measuring each side at least twice can one demonstrate that the differences between sides reflect actual asymmetry, rather than measurement error. The calculated average of the two measurements were used in the subsequent statistical analysis.

To preserve anonymity each participant received a unique ID at arrival, printed on A6 cards, and only this number was used to assign his/her measurements. Measurements of the three lower body traits were all performed using a digital Vernier caliper (Preisser products, Germany) to an accuracy of 0.01 mm. To eliminate potential measurement biases, the calipers were closed after each measurement. In addition to the three lower body traits, weight and height were measured and age was self-reported. Collecting all measurement took approximately 10 minutes per participant and there was a time span of some 3–5 minutes between first and second measurements of a trait, in which participants completed measurements at another station before they returned for the second measurement. Each measurement was called out to an assistant sitting next to the investigator to ensure that the investigator could not memorize them.

The size of the left and right knee of each participant was measured as defined by the largest breadth, measured between the medial and lateral epicondyles of the femur. The investigator measuring ankles first located the widest point of the ankle by hand before putting the caliper in place. For both knee and ankle measurements, participants were seated on a chair with legs bent by 90° and measurements were taken by an investigator sitting in front of the participant. Somatometric landmarks were palpated and measurements were taken with constant pressure of the caliper to minimize soft tissue related measurement error. Foot length was measured bilaterally using standardized A3 graphical paper (resolution of 1 mm) that was then measured with calipers. In a seated position, participants were instructed to place both feet on to the paper in a manner which aligned the pternion to the head of the 2nd metatarsal axes of each foot in parallel. Using a leveling device, markings were placed at the pternion and at the tip of toe one of each foot. To ensure consistency of measurement, markings were placed at the tip of toe one irrespective of whether toes two through five would have elicited a greater foot length. The perpendicular distance between two parallel lines extrapolated from the original foot markings was deemed foot length.

### Statistical analysis

Most analyses were performed using IBM SPSS Statistics 21. The presence of fluctuating and directional asymmetry (FA and DA) were assessed using the Excel template at www.biology.ualberta.ca/palmer/asym/FA/FA-Refs.htm#tools. With this template, the presence of significant FA (FA > measurement error) is demonstrated by a significant F-test for a sides X individuals interaction in a mixed model ANOVA [9], [10]. DA is indicated by a significant effect of sides in the ANOVA model.

Significant DA was present in feet and ankles (see Measurements in the main text). To ensure that statistical tests are comparing FA and are not biased by directional differences between sides, we used the index of FA developed by Graham et al. [11]. This index is based on unrotated factors extracted from the covariance matrix of a principal component analysis of the mean right side measurements on a trait and the mean left side measurements. This first factor represents the covariance between sides, plus half the FA and half the remaining measurement error. The second factor represents half the FA and half the remaining measurement error. This second factor is therefore an index of FA and does not include DA, which would be extracted with the covariance between sides. When analyzing overall asymmetry, including DA, we use relative asymmetry (absolute asymmetry in a trait divided by trait size).

Unsigned FA (absolute value of right – left) has an asymmetrical frequency distribution with only half of a bell curve. However, FA should not then be transformed to achieve normality, because F-tests are robust and transformation may change the underlying relationships between FA and other variables [10], [12]. Comparisons of FA are comparisons of variance in a trait, and comparisons of the means of two half-normal distributions gives an unbiased estimate of differences in variance [9], [10]. Parametric correlation and regression analyses are also robust in this case [12]. To avoid inflating Type I error by conducting multiple tests, multivariate analyses were used whenever appropriate. When the dependent variables were FA in individual traits, the three traits were tested simultaneously using multivariate GLM in SPSS (MANOVA, MANCOVA). When testing dependent variable composite FA (sum of the FA index for the three traits) we used univariate GLMs (ANOVA, ANCOVA). When the FA values were the independent variables and athletic performance (indexed by IAAF scores) the dependent variable, we used multiple regression.

Significance tests were conducted with an alpha level of 0.05, and, whenever appropriate, effect sizes are given in addition to p-values. Tests are two-tailed except when testing for relationships between FA and racing performance. In these situations we use a directed test, a compromise between one and two-tailed testing for strongly directional hypotheses [13], [14]. Our previous work [1] showed a positive relationship between symmetry (low FA) and sprinting and there is no reason to expect that greater symmetry would result in poorer performance, therefore we have a strong directional hypothesis. Our other comparisons are not as obviously directional: effects of weight, age, and gender on FA and on performance and effects of athletic training on FA.

The dataset is available in the Dryad repository (http://doi.org/10.5061/dryad.s3630).

### Ethical statement

This research was approved by the Institutional Review Board of Rutgers University, protocol #14–425 M. All participants were informed on the purpose of the study and gave written consent. Permission of legal guardians was also obtained for subjects below 18 years of age.

## Results

### Measurements

Mean values (+/−SD) for the three traits are as follows (all n = 189): right knee 96.80 mm+/−7.63; left knee 96.66+/−7.94; right ankle 67.99+/−5.40; left ankle 68.70+/−5.48; right foot 259.89+/−17.23; left foot 261.03+/−17.49. A Mixed Model Sides X Individuals ANOVA (see Methods) demonstrates significant FA in all 3 traits (FA > measurement error: knees F_188,378_ = 1.96; ankles F_188,378_ = 2.91; feet F_188.376_ = 5.55; all p<0.0001) and significant directional asymmetry (DA) in ankles (F_1,188_ = 34.76, p<0.0001) and feet (F_1,188_ = 23.00, p<0.0001) but not knees (F_1,188_ = 1.47, p = 0.23). The left foot and ankle tended to be larger than the right: 64.0% of subjects had larger left ankles and 61.9% had larger left feet, with little variation among groups. The significant p-values are so low that they are still at the p<0.0001 level even with any correction for multiple tests. Because of the presence of DA, we use FA values corrected for DA (see Methods).

### Athletes vs. controls

**Figure 1:**
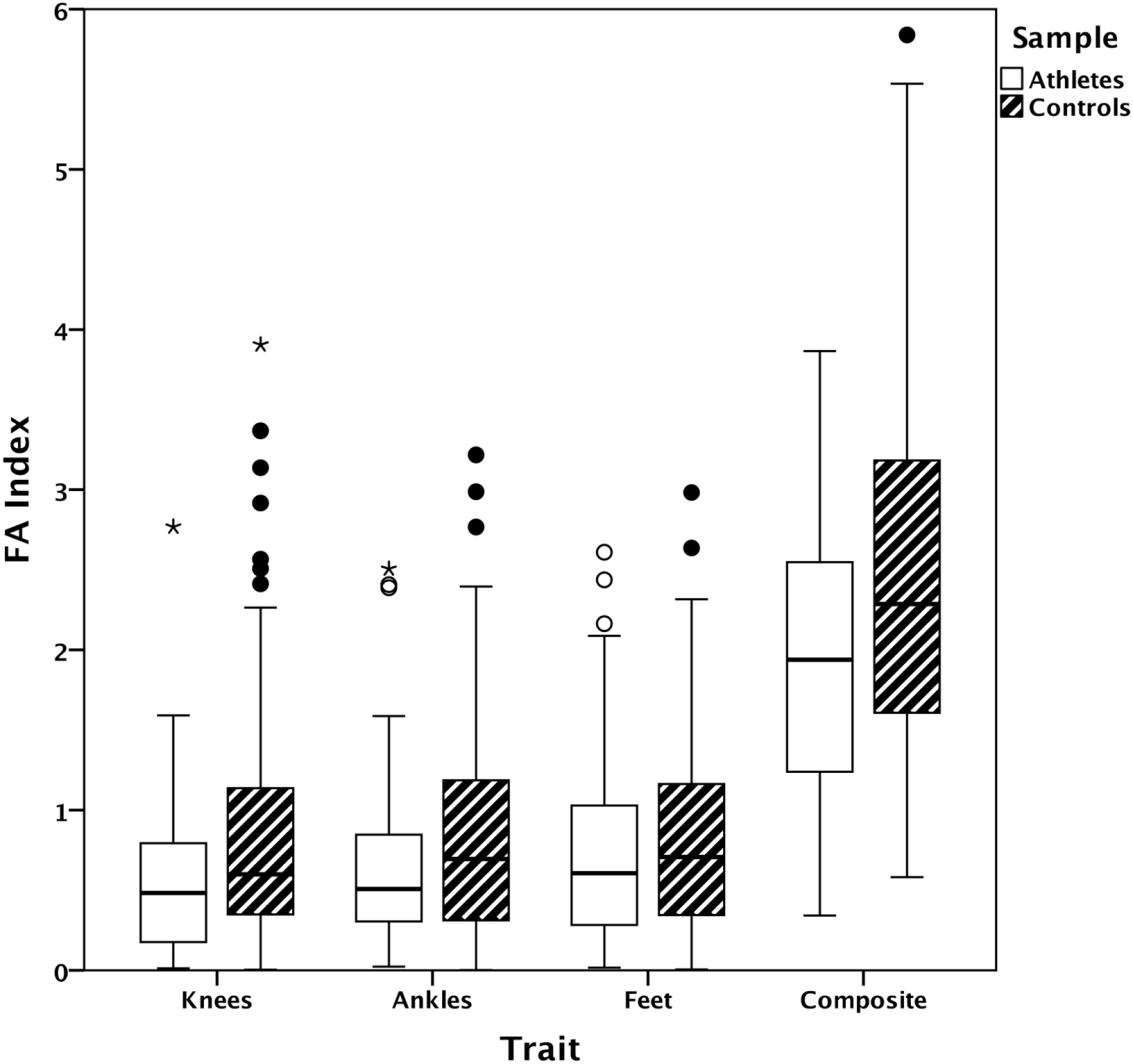
Boxplots for the three traits and their sum. FA is corrected for directional asymmetry using Graham et al.’s index (see Methods). Elite athletes (n = 73) are represented by open bars and controls (n = 116) by bars filled with diagonals. Circles represent outliers, with stars indicating extreme outliers; color of circles and stars corresponds to coloring in bars. Controls have significantly higher knee, ankle and composite FA than the athletes (see Tables 1–3).

Mean values of the FA index (corrected for DA) for athletes and controls are presented in Figure 1. The FA index was compared between elite athletes and controls in MANOVAs with dependent variables knee, ankle and foot asymmetry, and group (elite vs. control) and gender as the factors. There are significant differences between athletes and controls in knee and ankle asymmetry, but not in foot asymmetry (Table 1). There were no significant sex differences, but a significant gender x group interaction for knees because female controls had particularly high mean knee FA (mean+/−SD = 1.04+/−0.82, n = 56; compare to Figure 1). Using composite asymmetry (sum of knee, foot, ankle) rather than individual traits again demonstrates significant differences between athletes and controls and no significant effect of gender, but also no significant gender x group interactions (Table 2).

**Table 1:** Statistical comparisons of FA index between athletes and controls.

Treatment	Pillai’s Trace	F_3,183_	P	
Athlete vs. Control	0.083	5.55	0.001*	
Gender	0.013	0.83	0.48	
Gender X A v. C	0.041	2.59	0.055	
**Factor**	**Trait**	**F_1,185_**	**P**	**Partial eta^2^**
Athlete vs. Control	Knee	10.37	0.002*	0.053
Athlete vs. Control	Ankle	4.55	0.034*	0.024
Athlete vs. Control	Foot	0.62	0.43	0.003
Gender	Knee	2.24	0.13	0.012
Gender	Ankle	0.26	0.61	0.001
Gender	Foot	0.04	0.84	∼0
Gender X A. v. C.	Knee	4.60	0.033*	0.024
Gender X A. v. C.	Ankle	0.001	0.97	∼0
Gender X A. v. C.	Foot	3.13	0.079	0.017

Multivariate tests compare the three traits simultaneously (knee, ankle, foot). Statistically significant relationships (two-tailed tests, alpha = 0.05) are indicated with asterisks.

**Table 2:** Statistical comparisons of composite FA index (knee, ankle, and foot FA combined) between athletes and controls.

Factor	F_1,185_	P	Partial eta^2^
Athlete vs. Control	13.24	<0.0001*	0.067
Gender	0.54	0.46	0.003
Gender X A. v. C.	0.07	0.80	∼0
**Factor**	**F_1,183_**	**P**	**Partial eta^2^**
Athlete vs. Control	12.82	<0.001*	0.065
Gender	1.63	0.20	0.009
Gender X A. v. C.	0.002	0.96	∼0
Age	0.33	0.56	0.002
Weight	1.99	0.16	0.011

Results are shown first with no covariates (F_3,185_ = 4.86, p = 0.003, adjusted r^2^ = 0.058) and then with covariates age and weight added (F_5,183_ = 3.46, p = 0.005, adjusted r^2^ = 0.061). Statistically significant relationships (two-tailed tests, alpha = 0.05) are indicated with asterisks.

Including weight and age as covariates does not change the key results Again significant differences between athletes and controls are present for composite FA (Table 2) and separately for knees and ankles, but not feet (Table 3). The gender x group interaction is now significant for both knees and feet, rather than just knees, but not when using composite FA. There is also a positive relationship between weight and foot FA, but the overall relationship with weight is marginal and there are no significant relationships between age and FA (Tables 2,3).

**Table 3:** Statistical comparisons of FA index between athletes and controls, with age and weight added as covariates.

Treatment	Pillai’s Trace	F_3,181_	P	
Athlete vs. Control	0.083	5.43	0.001*	
Gender	0.023	1.43	0.24	
Gender X A v. C	0.046	2.94	0.035*	
Age	0.021	1.29	0.28	
Weight	0.036	2.23	0.087	
**Factor**	**Trait**	**F_1,185_**	**P**	**Partial eta^2^**
Athlete vs. Control	Knee	10.21	0.002*	0.053
Athlete vs. Control	Ankle	4.52	0.035*	0.024
Athlete vs. Control	Foot	0.49	0.48	0.003
Gender	Knee	2.26	0.14	0.012
Gender	Ankle	0.38	0.54	0.002
Gender	Foot	1.74	0.19	0.009
Gender X A. v. C.	Knee	4.34	0.039*	0.023
Gender X A. v. C.	Ankle	0.002	0.96	∼0
Gender X A. v. C.	Foot	4.50	0.035*	0.024
Age	Knee	0.13	0.72	0.001
Age	Ankle	0.88	0.35	0.005
Age	Foot	2.73	0.10	0.015
Weight	Knee	0.04	0.84	∼0
Weight	Ankle	0.05	0.82	∼0
Weight	Foot	6.57	0.011*	0.035

Multivariate tests compare the three traits simultaneously (knee, ankle, foot). Statistically significant relationships (two-tailed tests, alpha = 0.05) are indicated with asterisks.

### Within-athlete comparisons

Relationships between FA and performance of the athletes, measured by IAAF scores, were tested using multiple regression. For FA we used the residuals of a regression between FA corrected for DA and discipline, to control for effects of particular events on symmetry (see below). There is a significant negative relationship between composite FA and performance (i.e., more symmetrical athletes perform better; Table 4). Among individual traits, a significant relationship is present for knees, with ankles marginal (directional p = 0.087; Table 4). There is also a significant effect of gender. Even without considering covariates age and weight, the regression model explains 12 to 13% of the variance in performance (adjusted r^2^ = 0.12 when using composite FA and 0.13 when using individual traits). Partial r^2^ values indicate that knee or composite FA, depending on the model, are each related to approximately 5% of the variation in performance (Table 4).

**Table 4:** Multiple regressions within athletes, testing for relationships with performance (IAAF scores).

Factor	Beta	Partial r^2^	P
**Individual Traits**			
Resid. Knee FA	−0.22	0.05	0.040*
Resid. Ankle FA	−0.17	0.03	0.087
Resid. Foot FA	−0.09	0.01	0.28
Gender	0.31	0.10	0.007*
**Composite FA**			
Resid. Composite FA	−0.22	0.05	0.031*
Gender	0.33	0.11	0.004*

FA values are residuals of the FA index on the athletes’ primary events. Results are shown first for individual traits (F_4,68_ = 3.38, p = 0.014, adjusted r^2^ = 0.12) and then for composite FA (F_2,70_ = 6.20, p = 0.003, adjusted r^2^ = 0.13). P-values for the predicted relationship between FA and performance are directional (see Methods), and statistical significance is indicated by asterisks.

When age and weight are added in as covariates, there is a strong positive relationship between age and performance, and the regression model explains 27 to 28% of the variance in performance (adjusted r^2^ = 0.27 when using composite FA and 0.28 when using individual traits; Table 5). The relationship with composite FA remains significant and is related to 5% of the variation in performance, but relationships with weight and with individual traits are not significant (both knees and ankles are marginal; Table 5). The effect of gender is no longer significant, and likely resulted in part from a selection effect based on age: generally the most successful athletes are those most likely to continue competing as they age, and this may be especially true of women. Examination of the historical records of those six males and six females with the top ten finishes per sex (two of whom are in our sample) reveals that the age effect is probably not only due to selection–there is also typically a within-individual improvement with age for several years, but then a leveling off and a decline late in an athlete’s career, circa 28 years (www.iaaf.org/athletes).

**Table 5:** Multiple regressions within athletes, testing for relationships with performance (IAAF scores) with age and weight added as covariates.

Factor	Beta	Partial r^2^	P
**Individual Traits**			
Resid. Knee FA	−0.16	0.03	0.084
Resid. Ankle FA	−0.16	0.03	0.081
Resid. Foot FA	−0.06	0.01	0.34
Gender	0.21	0.02	0.20
Age	0.41	0.19	<0.0001*
Weight	−0.15	0.01	0.35
**Composite FA**			
Resid. Composite FA	−0.18	0.05	0.046*
Gender	0.24	0.04	0.12
Age	0.42	0.20	<0.0001*
Weight	−0.12	0.01	0.44

FA values are residuals of the FA index on the athletes’ primary events. Results are shown first for individual traits (F_6,66_ = 5.43, p<0.0001, adjusted r^2^ = 0.27) and then for composite FA (F_4,68_ = 8.04, p<0.0001, adjusted r^2^ = 0.28). P-values for the predicted relationship between FA and performance are directional (see Methods), and statistical significance is indicated by asterisks.

We also conducted analyses restricted to those whose best event is the 100 m sprint, because we predicted the relationship between FA and performance to be particularly important for sprinting events and our sample for sprinters, although small (n = 32), is larger than for any other events. Here we use the index of FA corrected for DA, but not the residuals with event, because we are testing within one event. A regression with dependent variable IAAF score and independent variables gender, knee, ankle, and foot FA is not significant (F_4,27_ = 1.97, p = 0.13, adjusted r^2^ = 0.11) but suggests relationships with knee and ankle FA (β = −0.33, directional p = 0.044 and β = −0.33, p = 0.045, respectively), but not foot FA (β = 0.18, p = 0.46) or gender (β = 0.10, two-tailed p = 0.56). The overall regression model is significant when covariates age and weight are added (F_6,25_ = 2.84, p = 0.030, adjusted r^2^ = 0.26), but the apparent relationships with knee and ankle FA are no longer significant (knees: β = −0.11, directional p = 0.34; ankles: β = −0.24, p = 0.093). For foot FA, β = 0.13, p = 0.55. Two-tailed p-values for the other variables are all greater than 0.05, but less than 0.10. Relationships with composite FA are not significant (directional p = 0.31 without covariates, 0.43 with covariates), because, within sprinters, foot FA trends opposite the predicted direction and therefore when knee and ankle FA are combined with foot FA the relationships become weaker. When using composite FA, significant effects of gender (β = 0.62, two-tailed p = 0.017), age (β = 0.39, p = 0.024), and weight (β = 0.55, p = 0.034) are all present and 25% of the variation in sprinting performance is explained (F_4,27_ = 3.56, p = 0.019, adjusted r^2^ = 0.25). IAAF scores should not show significant differences between males and females, because they are calculated to allow comparison of relative performance regardless of gender. The significant relationship we find is likely a result of our sample happening to contain particularly successful female sprinters.

**Figure 2:**
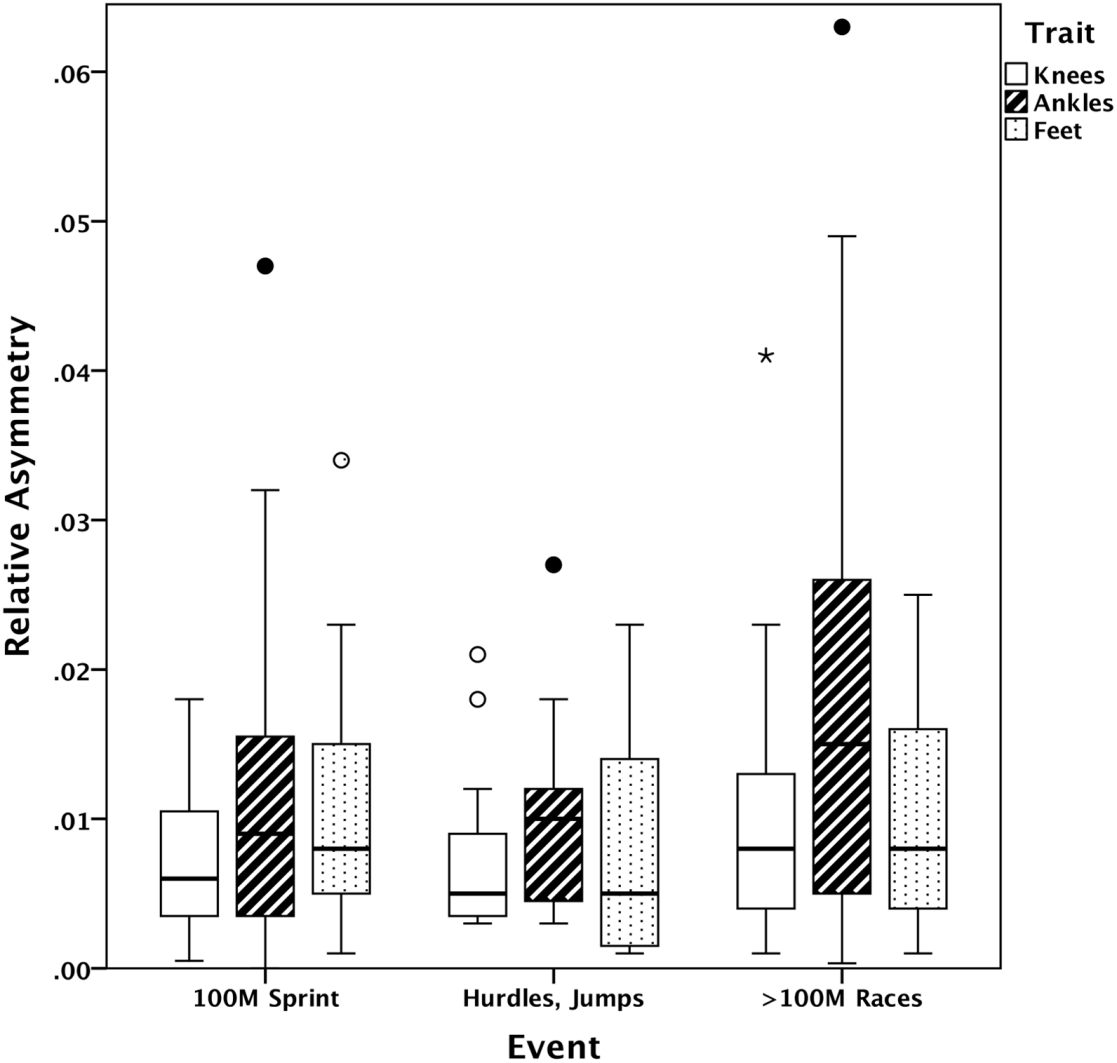
Boxplots for the three traits in athletes compared across events. These values reflect overall asymmetry, including FA and DA, divided by trait size. Knees are represented by open bars, ankles by bars filled with diagonals, and feet by bars filled with dots. Circles represent outliers, with stars indicating extreme outliers; color of circles and stars corresponds to coloring in bars.

In addition to testing for relationships between FA and performance, we tested whether symmetry among athletes varied with their primary event, because events differ in the stresses they produce and may cause both fluctuating and directional asymmetry. Because of small sample sizes for several events, the events were combined into three categories: straight sprints (100 m, n = 32), longer races with turns (200 m, 400 m, 800 m; n = 29), and events with jumping or leaping (100 and 110 m hurdles, 400 m hurdles, long jump, high jump; n = 11) [the one shot-putter was excluded]. An ANOVA test revealed significant variation among these categories in relative composite FA (not corrected for DA): F_2,69_ = 3.67, p = 0.031. A Tukey’s HSD post-hoc test revealed that longer distance runners were significantly more asymmetrical than sprinters. Means for sprinting and jumping events were similar. In a MANOVA, there were no significant differences among groups for the individual traits, but the trends for knees and especially ankles match the trend for composite FA (Fig. 2). If we use the FA index corrected for DA the patterns are similar, but are not significant (composite FA index, F_2,69_ = 2.37, p = 0.10). However, here we specifically want to test the effects of athletic stresses, which are often asymmetric, on overall symmetry and therefore DA should be included. Above we were interested instead in the relationships between developmental stability (indexed by FA) and performance, and thus needed to factor out DA.

## Discussion

Jamaicans are the elite sprinters of the world. Why? If symmetry of knees and ankles is a factor, why should Jamaicans be especially symmetrical (there is no knowledge of whether they actually are)? One possibility is heterozygosity for genes important to sprinting. The slave trade greatly increased heterozygosity on the West African side by mixing genes up and down the West coast of Africa from Senegal to Nigeria [15], [16]. Recently a mtDNA haplotype has been isolated that correlates with success in African American–but not Jamaican–sprinters [17]. Since there is a general (if often weak) positive relationship between heterozygosity and body symmetry [18] we are eager to do targeted studies of genomics on areas associated with sprinting, including energy substrate utilization, muscle fibre-type distribution and body composition analyses (with specific reference to the shape and size of the glutei maximi). Fast twitch (anaerobic) muscle fibres are characterized by specific adaptations which benefit the performances of explosive high-intensity actions such as those involved in sprinting. Notably, West Africans appear to have a higher fast twitch muscle fibre content than do comparable Europeans (67.5% vs 59% in one sample [19], as cited in [20]).

An interesting problem arises. How much is genetic quality revealed by overall body symmetry and how much is it associated with symmetry of particular body parts? To give a (partly) counterintuitive example, over-all body symmetry, based on 10 measurements of race horses, is positively correlated with performance in competitive races, but both lower body asymmetry, especially of the knees, is a correlate and so also are features of the head [21], as if the symmetry of the apparatus controlling the running (brain, head, sensory organs) is as important as the parts doing the running (themselves already selected for symmetry as shown in the Jamaican children, see [1]). Møller and colleagues [22] also demonstrated a link between lower-body symmetry and locomotion (in chickens), but did not measure upper-body traits. Manning and Pickup [23] showed for humans that degree of nostril symmetry positively predicts running performance in middle-distance runners, which is plausible since middle distance running relies on oxygen much more than do sprints and symmetrical organs of air intake should maximize consumption. Foot asymmetry predicts physical aggressiveness in boys [24] and lower body symmetry predicts the same for college undergraduates [25], perhaps because stability is especially important in fights. Ear asymmetry, in turn, predicts tendency for women and girls to cradle a baby or doll, respectively, on the right side [3]. By contrast, Tomkinson, Popović and Martin [26] failed to find any significant differences between elite football (soccer) and basketball players, nor between elite (national league) and sub-elite (state leagues) categories of each. This is not surprising given their methodology. Numerous traits, soft body and hard, were measured for symmetry in each individual, and then averaged together so that a single average value was typically used. In our own work, symmetry of one part of the body (knees) suggests quality of associated parts as well, not just the bones of the knee but the attached cartilage, muscle, associated structures and developmental control. We know nothing about the details of this. The only part of the sprinting apparatus thought in advance to be important that we did not measure was the buttocks, hips, and glutei maximi (the largest and strongest muscles in the human body). Muscular strength measurements and imaging techniques such as dual-energy X-ray absorptiometry may allow a chance for progress on this (possibly key) variable.

A second problem has to do with cause and effect. If we find that those elite sprinters with the best times also have the most symmetrical knees, is it because those with symmetrical knees do well in advance or is it that intensive training leads to both success and more symmetrical knees? Of course it is likely that cause and effect go in both directions, and there is a weak trend toward decreased knee FA with age in the athletes (r = −0.15, p = 0.22). However, the fact that 8 year old knee FA predicts sprinting ability 14 years later [1] and that there are no statistically significant changes in symmetry with age in elite athletes, even though the older will have exposed themselves to symmetrical forces during training longer than the younger, suggests that knee symmetry is by no means a mere reflection of training.

In addition to the positive effects of symmetry on running performance that we demonstrate, we also find differences in symmetry between running events. Runners specializing in longer races are less symmetrical than sprinters, and this difference is particularly noticeable for the ankles. Why this is the case is unclear, but may relate to the asymmetrical stresses of events requiring turns and an increased prevalence of rearfoot striking in longer events, which would alter the distribution of stresses acting on the joints [27].
